# Supplementary material for: Occurrence, Concentration and Toxicity of 54 Polycyclic Aromatic Hydrocarbons in Butter during Storage
Source: Foods. 2023 Dec 6;12(24):4393. doi: 10.3390/foods12244393 (PMC10742937; doi:10.3390/foods12244393)
Supplement: Supplementary file 1 [file foods-12-04393-s001.zip › foods-2749563-supplementary.pdf]

Supporting Information

Occurrence, Concentration and Toxicity of 54 Polycyclic Aromatic Hydrocarbons in Butter during Storage

Jianqiang Lan, Shimin Wu*

Department of Food Science and Technology, School of Agriculture and Biology, Shanghai Jiao Tong University, 800 Dongchuan Road, Shanghai 200240, China

* Corresponding author: Tel./Fax: +86 21 34205717; E-mail: wushimin@sjtu.edu.cn

Table S1 Nutrition facts for 21 butter types and 5 margarine types

Sample	Fat (g/100g)	Protein (g/100g)	Carbohydrate (g/100g)	Na (mg/100g)	Shelf life (Mon)
B1(AN*)	82.0	0.7	1.0	12	12
B2(AY)	80.0	0.7	1.0	800	12
B3	82.0	0.7	0.9	20	12
B4	80.0	0.7	0.9	750	12
B5	82.0	0.7	0.8	20	12
B6(BN)	82.9	0.6	0.6	10	18
B7(BY)	81.4	0.6	0.6	600	20
B8	82.8	0.0	0.0	11	24
B9	81.6	0.0	0.0	531	24
B10	82.0	0.7	0.6	0	12
B11(CN*)	82.1	0.6	0.6	0	24
B12	82.0	1.0	1.0	0	24
B13(EN*)	82.0	0.6	0.6	8	18
B14(EY)	80.0	0.6	0.6	730	18
B15(DN)	82.1	0.6	0.6	0	24
B16(DY)	81.0	0.6	0.6	550	24
B17	82.0	0.7	0.6	16	12
B18	96.0	0.0	0.9	268	12
B19	99.8	0.0	0.0	40	12
B20	99.8	0.0	0.0	40	12
B21	83.0	0.6	0.6	10	12
M1	82.0	0.0	0.0	590	12
M2	83.0	0.0	0.0	500	12
M3	81.0	0.0	0.0	179	12
M4	81.0	0.0	0.0	0	12
M5	84.0	0.0	1.2	0	12

Table S2 Experimental conditions of MRM modes of GC-QqQ-MS for 24 PAHs.

PAHs	RT (min)	Quantitative ion (m/z)	Collision energy (eV)	Qualitative ion (m/z)	Collision energy (eV)
Nap	4.076	128.0>102.0	25	128.0>78.0	30
Ap	6.157	152.0>151.0	25	152.0>126.0	25
Ac	6.331	154.0>153.0	20	153.0>152.0	25
F	7.072	166.0>165.0	25	166.0>115.0	40
Phe	9.364	178.0>152.0	25	178.0>177.0	20
Ant	9.448	178.0>152.0	25	178.0>177.0	20
Flu	13.364	202.0>200.0	40	202.0>201.0	30
Pyr	14.477	202.0>200.0	40	202.0>201.0	30
BcF	16.143	216.0>215.0	30	216.0>190.0	35
BaA	20.61	228.0>226.0	40	226.0>225.0	35
CP	20.906	226.0>225.0	35	226.0>224.0	40
Chr	21.093	228.0>226.0	40	226.0>225.0	35
5-MChr	23.789	242.0>241.0	20	242.0>215.0	25
BbF	27.516	252.0>250.0	40	252.0>226.0	40
BkF	27.697	252.0>250.0	40	252.0>226.0	40
BjF	27.849	252.0>250.0	40	252.0>226.0	40
BaP	30.031	252.0>250.0	40	252.0>226.0	40
IP	36.424	138.0>137.0	20	276.0>275.0	40
DahA	36.606	278.0>276.0	40	138.0>137.0	20
BghiPE	37.983	276.0>274.0	40	276.0>275.0	40
DBalP	43.179	302.0>300.0	40	302.0>301.0	30
DBaeP	44.959	302.0>300.0	40	302.0>301.0	30
DBaiP	46.064	302.0>301.0	30	302.0>300.0	40
DBahP	46.72	302.0>300.0	40	302.0>301.0	30

Experimental conditions of MRM modes of GC-QqQ-MS for other PAH derivatives were provided in our previous study [1].

Table S3 Concentrations of PAH24 in butter and margarines (μg/kg)

Sample	Nap	Ap	Ac	F	Phe	Ant	Flu	Pyr	BcF	BaA	Chr	BbF	BkF	BjF	BaP	DahA	BghiPE
B1(AN*)	60.2±0.1	6.8±0.3	1.25±0.06	6.19±0.02	25.5±0.5	2.01±0.01	12.2±0.4	38.95±0.01	2.47±0.00	2.05±0.01	4.12±0.04	1.05±0.03	0.45±0.01	0.68±0.01	1.31±0.01	n.q.	0.38±0.03
B2(AY)	80.6±0.3	6.8±0.2	1.06±0.07	5.07±0.06	23.5±0.6	n.q.	12.0±0.2	47.5±0.2	1.94±0.03	1.26±0.01	n.q.	0.93±0.02	0.44±0.02	0.60±0.00	1.23±0.02	0.47±0.01	0.3±0.1
B3	51.7±0.4	10.6±0.6	1.15±0.00	5.7±0.2	25.62±0.03	1.87±0.01	8.8±0.2	41.9±0.6	1.57±0.00	1.00±0.02	n.q.	0.69±0.01	0.35±0.01	0.56±0.00	1.15±0.00	n.q.	0.29±0.00
B4	40.37±0.01	9.3±0.3	1.5±0.4	5.8±0.1	26.09±0.06	2.39±0.04	12.9±0.2	39.2±0.3	1.89±0.01	0.91±0.05	n.q.	0.69±0.00	0.35±0.00	0.55±0.01	1.1±0.1	n.q.	n.q.
B5	45.0±0.3	8.5±0.5	1.17±0.07	4.8±0.4	13.3±0.3	n.q.	6.1±0.2	25.56±0.09	1.31±0.04	0.95±0.03	n.q.	0.69±0.00	0.34±0.00	0.55±0.00	1.05±0.00	n.q.	n.q.
B6(BN)	47.1±0.1	12.9±0.5	1.17±0.08	5.2±0.2	21.1±0.5	2.27±0.08	11.45±0.02	31.1±0.2	1.92±0.01	1.64±0.01	0.37±0.00	1.03±0.01	0.43±0.01	0.67±0.00	1.25±0.02	n.q.	0.31±0.00
B7(BY)	77.3±0.5	7.7±0.7	1.53±0.03	5.37±0.04	22.8±0.1	n.q.	12.37±0.02	42.0±0.7	1.7±0.1	1.37±0.03	n.q.	0.91±0.01	0.42±0.01	0.62±0.00	1.22±0.02	n.q.	0.30±0.00
B8	34.62±0.09	6.45±0.02	0.92±0.06	3.3±0.1	14.4±0.2	1.06±0.04	4.6±0.1	4.2±0.2	1.43±0.04	0.93±0.01	n.q.	0.66±0.00	n.q.	0.54±0.00	1.03±0.01	n.q.	n.q.
B9	18.4±0.3	9.4±0.5	1.69±0.03	5.7±0.2	21.63±0.04	n.q.	6.5±0.2	6.02±0.07	1.38±0.03	1.01±0.00	n.q.	0.70±0.01	0.36±0.01	0.56±0.00	1.01±0.01	n.q.	n.q.
B10	15.5±0.2	11.33±0.04	0.87±0.06	4.43±0.02	14.6±0.2	n.q.	3.61±0.07	3.67±0.00	1.41±0.02	0.97±0.00	n.q.	0.74±0.00	0.39±0.00	0.56±0.01	1.04±0.01	n.q.	n.q.
B11(CN*)	78.6±1.0	10.5±0.7	1.87±0.09	9.9±0.2	45.5±0.2	n.q.	22.18±0.08	55.50±0.07	2.7±0.4	1.68±0.02	6.9±0.2	1.10±0.03	0.46±0.02	0.67±0.00	1.60±0.00	n.q.	0.4±0.1
B12	18.01±0.06	10.5±0.2	3.73±0.09	18.7±0.3	70.2±0.3	n.q.	10.40±0.03	11.3±0.2	3.1±0.2	1.29±0.00	1.8±0.1	0.68±0.00	0.39±0.01	0.74±0.00	n.q.	n.q.	n.q.
B13(EN*)	131.86±0.00	15.4±0.5	1.26±0.02	11.0±0.3	42.3±0.3	1.9±0.2	17.7±0.3	75.8±0.3	2.38±0.08	1.30±0.05	5.3±0.1	1.69±0.00	0.43±0.02	0.62±0.00	1.34±0.00	n.q.	0.42±0.01
B14(EY)	73.27±0.04	4.6±0.3	0.50±0.02	1.87±0.07	10.43±0.01	1.00±0.01	7.6±0.1	45.8±0.5	1.27±0.05	1.11±0.06	n.q.	0.83±0.03	0.43±0.02	0.58±0.00	1.14±0.01	n.q.	0.33±0.00
B15(DN)	14.3±0.3	17.2±1.3	2.8±0.2	16.0±0.1	69.1±0.2	n.q.	4.1±0.2	5.1±0.2	1.32±0.01	0.94±0.06	1.26±0.08	0.71±0.02	0.38±0.00	0.74±0.00	n.q.	n.q.	n.q.
B16(DY)	129.4±0.2	20.8±1.1	1.32±0.03	7.6±0.7	31.1±0.1	n.q.	19.7±0.3	68.3±0.7	2.69±0.05	2.63±0.01	1.78±0.03	1.04±0.03	0.51±0.00	0.75±0.00	1.47±0.01	n.q.	0.37±0.00
B17	19.42±0.01	3.0±0.3	0.41±0.03	1.24±0.01	2.58±0.04	0.32±0.00	4.9±0.3	20.9±0.3	1.12±0.02	0.81±0.02	n.q.	0.69±0.00	0.36±0.01	0.55±0.00	1.04±0.01	n.q.	n.q.
B18	14.92±0.05	2.3±0.1	0.42±0.01	1.18±0.04	7.6±0.2	0.99±0.01	4.6±0.4	13.4±0.2	1.73±0.01	0.91±0.04	n.q.	0.67±0.00	0.35±0.01	0.55±0.00	1.03±0.02	n.q.	0.17±0.01
B19	18.2±0.3	3.93±0.04	0.52±0.01	3.56±0.08	11.42±0.09	0.95±0.00	5.7±0.1	16.3±0.6	2.25±0.03	1.37±0.04	n.q.	0.79±0.00	0.38±0.00	0.60±0.00	1.13±0.03	n.q.	n.q.
B20	43.0±0.6	6.90±0.02	0.51±0.01	1.50±0.03	8.50±0.06	0.43±0.01	6.9±0.1	40.9±0.1	2.26±0.03	1.02±0.03	n.q.	0.73±0.00	0.37±0.00	0.57±0.00	1.02±0.02	n.q.	n.q.
B21	38.3±0.7	8.8±0.5	1.0±0.2	5.9±0.1	25.31±0.02	1.61±0.05	7.90±0.01	30.26±0.07	1.34±0.07	0.84±0.02	n.q.	0.67±0.01	0.35±0.00	0.55±0.00	1.04±0.02	n.q.	0.23±0.01
M1	63.8±0.2	7.2±0.5	0.96±0.05	6.03±0.09	6.68±0.04	2.25±0.04	8.7±0.3	17.2±0.2	1.49±0.05	1.14±0.02	n.q.	0.84±0.04	0.36±0.00	0.61±0.00	1.16±0.05	n.q.	0.22±0.02
M2	14.1±0.5	2.9±0.2	0.62±0.07	2.72±0.07	16.4±0.3	n.q.	3.08±0.00	3.00±0.08	1.72±0.02	1.26±0.06	n.q.	0.86±0.04	0.45±0.01	0.59±0.00	1.04±0.02	n.q.	0.16±0.01
M3	19.13±0.04	5.2±0.2	0.37±0.01	0.51±0.01	0.82±0.00	0.48±0.02	3.7±2.0	12.48±0.04	0.92±0.03	1.07±0.04	n.q.	0.97±0.04	0.47±0.04	1.13±0.08	0.44±0.04	n.q.	n.q.
M4	16.2±0.4	6.40±0.03	0.32±0.01	0.09±0.00	1.19±0.02	0.39±0.04	2.60±0.02	13.08±0.05	1.41±0.01	0.99±0.05	n.q.	1.05±0.03	0.41±0.01	1.01±0.02	0.64±0.00	n.q.	n.q.
M5	27.8±0.2	10.6±0.2	0.61±0.01	1.07±0.04	5.0±0.1	0.72±0.00	6.5±0.1	39.1±0.2	2.56±0.04	1.56±0.08	0.16±0.00	1.11±0.07	0.50±0.00	1.17±0.08	0.73±0.05	n.q.	n.q.

n.q., not quantified; None of 5-MChr, IP, CP, DalP, DaeP, DaiP, and DahP were quantified.

Table S4 OSI of nine types of butter (h)

Sample	AN*	AY	BN	BY	CN*	DN	DY	EN*	EY
OSI	1.59	1.64	1.77	1.81	1.60	1.01	1.74	1.99	1.81

1. Li, W.; Wu, S. Halogenated polycyclic aromatic hydrocarbons and their parent compounds in ready-to-eat seafood rich in salt: Method validation, profiles, correlation, and exposure risks. *Food Control* **2022**, *136*, doi:10.1016/j.foodcont.2022.108864.
